# Challenges of Testing COVID-19 Cases in Bangladesh

**DOI:** 10.3390/ijerph17186439

**Published:** 2020-09-04

**Authors:** Khan Rubayet Rahaman, Md. Sultan Mahmud, Bishawjit Mallick

**Affiliations:** 1 Department of Geography and Environmental Studies, St. Mary’s University, Halifax, NS B3H 3C3, Canada; khan.rahaman@smu.ca; 2 United Nations, Planning Unit, Shelter and Site Division, Cox’s Bazar 4700, Bangladesh; sultan.mahmud@udo.edu; 3 Chair of Environmental Development and Risk Management, Faculty of Environmental Sciences, Technische Universität Dresden (TUD), 01217 Dresden, Germany; 4 Marie Curie Global Fellow at Institute of Behavioral Science, University of Colorado Boulder, Boulder, CO 80302, USA

**Keywords:** COVID-19, environment, decentralisation, Bangladesh, public health, policies, management

## Abstract

Keeping the dynamic nature of Coronaviruses (COVID-19) pandemic in mind, we have opted to explore the importance of the decentralization of COVID-19 testing centers across the country of Bangladesh in order to combat the pandemic. In doing so, we considered quantitative, qualitative, and geographic information systems (GIS) datasets to identify the location of existing COVID-19 testing centers. Moreover, we attempted to collect data from the existing centers in order to demonstrate testing times at the divisional level of the country. Results show that the number of testing centers is not enough to cater to the vast population of the country. Additionally, we found that the number of days it takes to receive the results from the COVID-19 testing centers is not optimal at divisional cities, let alone the remote rural areas. Finally, we propose a set of recommendations in order to enhance the existing system to assist more people under a testing range of COVID-19 viruses at the local level.

## 1. Introduction

As global cases of the novel coronavirus disease (COVID-19) exceed 10 million with a confirmed global death toll of more than 509,779 (Until 30 June, 2020) people [1], scientists emphasize the critical need to escalate testing, isolation, contact tracking efforts, and build awareness among communities in order to respond to the pandemic [2,3,4,5,6]. Governments around the world are responding immediately to this novel virus pandemic with unprecedented policies in order to slow the growth rate of infections [7,8,9,10]. Note that the World Health Organization (WHO) on 11 March, 2020, declared the novel coronavirus (COVID-19) outbreak a global pandemic [11,12]. Moreover, the WHO recommended that people with mild respiratory symptoms should be encouraged to isolate themselves, and social distancing is emphasized and may apply even to countries with no reported case [11]. Despite the drastic, large-scale containment measures promptly implemented by governments across the world, in a matter of few weeks, the disease spread to most of the countries in the world [13,14,15,16,17,18,19,20,21,22]. As a result, policymakers are opting to enhance testing numbers rapidly in order to identify infected patients immediately so that public health measures can be taken into consideration. South Korea has introduced a large organized testing program, combined with extensive efforts to isolate infected people and trace and quarantine their contacts [7,23,24].

Consequently, the country has introduced COVID-19 testing opportunities for local people with a network of a considerable number of drive-through testing stations [24]. However, several developed countries such as USA, Italy, France, and the UK have been plagued by an overly bureaucratic system, and problems with its test kits have had a slow start [15,16,22,24,25,26]. In Europe, Germany is a front-runner with more than 100,000 tests processed per week [24]. In this situation, countries cannot fight this pandemic without identifying where the cases are. Michael Salathe, a computational epidemiologist at the Federal Institute of Technology of Lausanne, stated, ‘at this point, 100% of nations got it under control, did so, based on testing and tracing, isolation, quarantine [24].’ However, many countries do not have the capacity and necessary resources in order to take necessary measures for setting up testing points in their jurisdiction.

Despite being a developing country, Bangladesh has taken some vital steps in the health sector. Significantly, the reduction of infant mortality, maternal care, and immunization are the key steps to ensure healthcare for all. The country has made remarkable progress in these sectors over the last two decades [27,28]. However, Bangladesh, one of the most populous countries in the world with a density of 1104 persons per sq. km, is experiencing quick spread of COVID-19 after the first case was identified on 8 March, 2020 [29,30,31]. To date, until June 30, 2020, the total reported case of COVID-19 in the country is 149,242, with a death toll of 1888 [1]. Such information hints that coronavirus has widely spread in the country [32]. Large scale testing allows health services to quickly identify who has the disease and arrange for them to receive the care needed. Moreover, it helps to isolate the known cases to prevent them from coming into contact with others and slows the rate of community spread or transmission. Effective testing programs around the world allow governments and health authorities to understand how prevalent the disease is and how it is evolving.

Although the country has made remarkable progress in health sectors, considerable challenges remain in the forefronts in the efforts to improve the health status of the population, including the health services inequalities, the quality of care and public satisfaction with healthcare, and the efficiency and sustainability of service-providing agencies [27]. These challenges point to the growing need for relevant and applied research to increase knowledge about the role of planning and stakeholders in the healthcare system. Consequently, tracking COVID-19-positive test results helps authorities make evidence-based decisions in order to take measures for slowing down the spread of the disease. Government policies about testing all people with possible coronavirus infections have widely varied from country to country. However, several countries around the world have identified symptoms that may lead to COVID-19 infections. These symptoms may appear 2–14 days after exposure to the virus as described in several documents, such as fever or chills, cough, shortness of breath or difficulty breathing, fatigue, muscle or body aches, headache, loss of taste or smell, sore throat, congestion or runny nose, nausea or throwing up, and diarrhea [2,5,9]. 

Depending on the testing outcome, a critical decision relates to whether patients are considered for enrollment in hospitals or health centers for possible treatment. In addition, testing is essential to know if the patient requires emergency medical attention when considering symptoms such as trouble breathing, persistent pain or pressure in the chest, inability to wake or stay awake, and bluish lips or face [2,5,9,33]. Moreover, generic approaches are recommended to combat community spread of the COVID-19 virus by following some generic approaches such as the following: (i) maintaining a physical distance of 6 feet (2 m); (ii) covering the face when coughing or sneezing with a tissue, disposing of the tissue, and handwashing; (iii) wearing a cloth face covering over the nose and mouth when visiting public areas; (iv) avoiding touching the eyes, nose, and mouth; (v) staying at home when the identified person is sick, except when seeking medical care; and (vi) washing hands often with soap and water for at least 20 s [26,34,35,36,37]. However, in a densely populated country like Bangladesh, testing plays a critical role in perceiving spatial distribution of the COVID-19 virus and taking necessary measures immediately. It is worth noting that if there are not enough tests, symptomatic patients should be prioritized as they may spread the virus to others in the community.

At this point, the question of whether Bangladesh is prepared to test a sufficient amount of the population while covering geographical locations in the whole country with the available resources and facilities in healthcare support services arises. This means that the number of facilities currently available in the health sector can be used to test people in all remote areas of the country, and this study examines the disadvantages of doing so. Regardless, it is critical to evaluate if the testing centers are spatially distributed proportionately in relation to the populations across the country in order to confirm accessibility for COVID-19 testing opportunities for all. Moreover, it is essential to understand the testing capacity and time requirements for serving a huge population at the local government level (i.e., district and sub-district level) across the country. As a result, this study opts to explore the operational number of COVID-19 testing centers across the country, and whether it may serve a sufficient amount of the population throughout the country in order to gain an understanding of the number of infected people. Consequently, the study takes an attempt to identify how many centers are capable of publishing the results in the same day so that isolation and quarantine can be possible immediately for imposing a “lock-down” requirement if necessary. With the outcome of this result, decision-makers at central and local governments may take immediate measures to collaborate with different stakeholders in order to combat this pandemic.

## 2. Materials and Methods

### 2.1. Study Area

**Figure 1:**
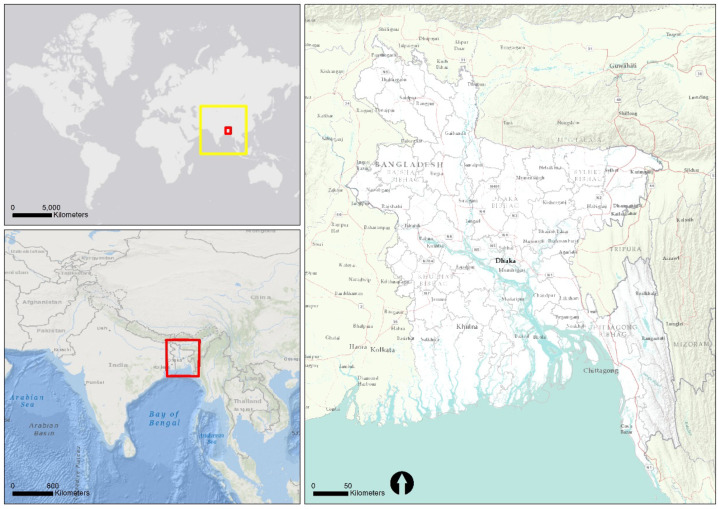
Map depicting the country boundary of Bangladesh in South Asia.

Bangladesh is a deltaic developing country located in South Asia [38]. The country is relatively small, with a land area of 147,570 km^2^ having the 8th largest population in the world and the 13th highest population density [39]. Figure 1 demonstrates the location of Bangladesh in South Asia. According to the UN (United Nations) report, the population of the country will further increase to 186 million and 202 million by the years 2030 and 2050, respectively [40]. Agricultural practices in the economy still dominate in Bangladesh since it gained independence in 1971. Because the unique location of the country is a low-lying deltaic floodplain, over one-fifth of the land of Bangladesh is flooded for approximately half a year, and sometimes as much as two-thirds of the land becomes uninhabitable during years when riverine flooding occurs due to extreme weather events [41]. Additionally, the country is recognized as one of the most vulnerable countries to climate change in the world due to a highly variable environment [4]. The rural population of the country consists of almost two-thirds of the total population [41]. Across the country, migration and mobility have become an integral part of rural livelihood strategies [42,43]. For instance, it is difficult to restrict people’s movement during the pandemic, as this may enhance vulnerability to a pro-poor segment of the population. Despite the growth of population, economy, and rapid urbanization, Bangladesh has been significantly susceptible to several contagious diseases in recent history, with dengue, malaria, and cholera taking thousands of lives [41,44,45].

The governance structure of the country has been decentralizing since the 1980s. The present structures consist of several major divisions, each of which is subdivided into several districts. These districts are divided further into smaller units called sub-district. Right now, Bangladesh is comprised of 8 divisions, more than 64 districts, and around 500 sub-districts [46].

Decentralization approaches have also been introduced in the healthcare system. Notably, the health centers are distributed throughout the country at sub-district levels. In an attempt to reach grassroot people at the local government level after independence, Bangladesh introduced several laws, plans, and policies in the health sectors with several reforms and alternatives [7]. The Ministry of Health and Family Welfare of Bangladesh has been the key player in improving health sectors with diverse policy frameworks over the last two decades. Under this ministry, separate councils were formed to regulate medical practitioners and providers, dentists, nurses, medical attendees, and diagnostics [7]. Importantly, in Bangladesh, healthcare is provided through public and private hospitals or privately run clinics.

Furthermore, the country still lags far behind in providing healthcare to the rich as well as the poor. In recent years, neighboring countries India and Thailand have come a long way in developing the skills and experience of physicians, improving healthcare technology, and developing high-quality hospitals and health management institutions [28]. That is why many people in Bangladesh go to India or Singapore for healthcare. According to the World Bank (2018), there are only 0.5809 physicians, 0.476 community health workers, 0.4124 nurses and midwives, and 0.8 hospital beds per 1000 people in Bangladesh. 

However, the demand for exerting pressure due to COVID-19 pandemic has urged the health sector to respond to diverse challenges right now. A recent study claims that until 26 March, 2020 the Institute of Epidemiology, Disease Control and Research (IEDCR) was the sole institute in Bangladesh with the testing facilities for COVID-19, and this single and under-resourced institute was unable to tackle the wave of suspected COVID-19 patients [47]. Due to such limited resources, timely testing of symptomatic patients was not possible, and the government has still not sought to limit community transmission from primary cases proactively. However, with a population of 161 million and a total of 1169 intensive care unit (ICU) beds, this inadequate strategy could potentially devastate Bangladesh’s health system with multiple outbreaks [48]. Thus, this study considers testing facilities and its spatial distribution in the country in order to assess what other facilities need to be ensured for tackling the COVID-19 pandemic in Bangladesh.

### 2.2. Data and Analysis

We opted to use qualitative, quantitative, and geographic information systems (GIS) data in order to obtain results. Issues of social opportunity can be easily presented through a combination of geographical and social information. As a result, the geographically unequal distribution of services is accurately represented, and equality may be ensured during future infrastructure and service planning. However, we understood the limitations of the expected data because of the pandemic and its unique characteristics in Bangladesh. Despite these facts, we highly relied on authentic sources of information, and scientifically utilized the data by considering the types and sources in the following subsections.

#### 2.2.1. COVID-19-Related Quantitative Data

The data relevant to COVID-19 were initially obtained from several reliable government websites. The number of cases was regularly updated in the Coronavirus Covid-19 Dashboard 2020, the site maintained by the government of Bangladesh. Moreover, data related to the number of testing centers, the number of medical staff, the number of infected people per day, and the number of death per day were obtained from several websites. Note that we crosschecked most of the data utilizing several sources, e.g., newspapers, print media, social media, and websites of international organizations such as John Hopkins University’s dashboard and the WHO. Consequently, we reviewed contemporary literature (i.e., published and unpublished), newspaper articles, internet blogs, opinion pieces, and local newsletters in order to obtain information with particular emphasis on Bangladesh.

#### 2.2.2. Qualitative Information

Under this pandemic, it was challenging to meet professionals and managers working at the field level in COVID-19 testing centers. However, we prepared a list of possible contact persons in several district-level offices who were engaged in the testing systems. Once we prepared the list, we then collected the telephone numbers of the people we were interested in contacting. Afterwards, we contacted the relevant persons to talk over the phone (on average for 15 min). Note that, because of some ethical agreements before consulting over the phone, we were not in a position to ask questions outside of our research topics, although a wide range of information was collected. Consequently, we collected qualitative information from the “talk-shows” regularly organized in the television media, emphasizing the testing capabilities and capacities of centers/labs situated across the country. However, these unstructured phone conversations mainly attempted to capture information related to the number of tests per centers and associated official procedures.

**Figure 2:**
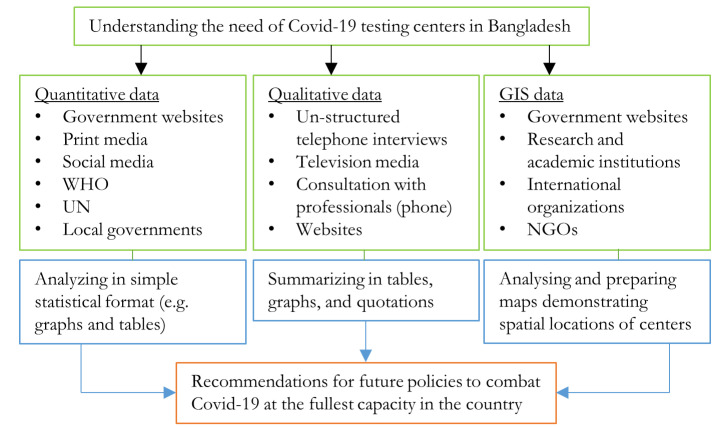
Schematic diagram of the study.

Figure 2 exhibits the schematic diagram of the methods adopted in this study. Note that the recommendations were made after the critical evaluation of the available information.

#### 2.2.3. GIS Data

We collected the GIS datasets from different sources including but not limited to the following: government websites; GIS labs from academic and research institutions (i.e., universities); non-government organizations (NGOs); and international organizations’ webpages (e.g., United Nations). Note that the necessary data were projected appropriately. Hence, we opted to clean the obtained datasets (i.e., *.shp, *.dbf files) and projected the data using WGS84 reference ellipsoid systems. Moreover, we plugged the COVID-19-related database in the GIS systems to obtain locations of testing centers in the country. In addition, we used ArcGIS software (developed and licensed by Environmental Systems Research Inc.) in order to analyze information and to generate the maps.

## 3. Results

### 3.1. Dynamics of the COVID-19 Situation in Bangladesh

**Figure 3:**
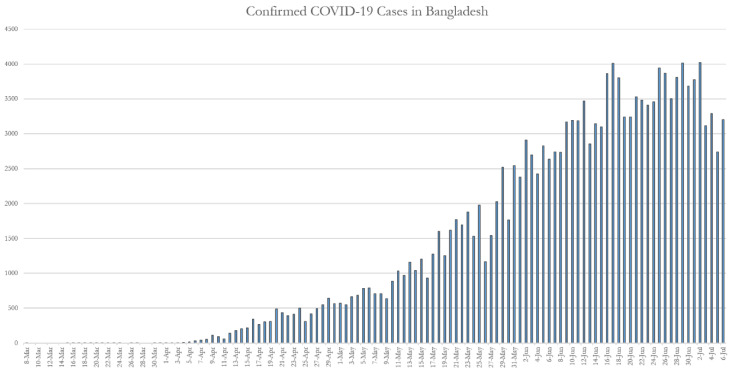
Confirmed COVID-19 cases up to 6 July 2020, (Source: Corona Virus Dashboard, Bangladesh). COVID: coronavirus disease.

The most popular argument regarding COVID-19 virus bearers is in relation to people who come from abroad to the country by international flights. Since its outbreak in December 2019 at Wuhan in China, Bangladesh has screened over 650,000 people in its all international airports, ports and land borders, although there were only 28,483 people in quarantine and 47 in isolation as of 28 March. As a result, the COVID-19 situation was not too severe until the end of March 2020 in Bangladesh. The first COVID-19 case was identified in the country on 8 March 2020 (i.e., three cases were found). Later, the situation has deteriorated day by day, and by 6 July 2020 there was a total of 165,618 confirmed case. Figure 3 shows an incremental trend of confirmed cases, and there is hardly any sign of flattening in the infection curve. The first thousand cases (i.e., 1034 on a single day) were reported on 11 May 2020, and this figure increased up to 4019 cases by 2 July 2020. The number of confirmed cases was increasing because the outbreak of the virus reached the remote small towns and villages. Moreover, the government arranged sample collection facilities in all government-affiliated hospitals, both in cities and rural health complexes. 

Again, the first death due to COVID-19 virus was on 18 March 2020 in Bangladesh, and the COVID-19 related death rate increased by 70% from 18 March 2020 to 6 July 2020. A recent total death count due to COVID-19 is 2096. Available data demonstrate that the number of infected COVID-19 patients has been increasing very rapidly, and several groups of people are susceptible to being infected, such as elderly people and patients with diabetes, heart disease, and kidney-related complications. Thus, it is important to provide necessary medical support for these patients with the highest priority in testing opportunities.

Additionally, it is also critical to keep in mind that in terms of tackling a disease outbreak, Bangladesh ranked the worst amongst the South Asian Countries in the 2019 Global Health Security Index. The US-based Johns Hopkins University developed this index. However, the country has undertaken several safety measures, including temporarily closing the international borders, and cancelling visa-on-arrival services at significant gateways.

### 3.2. Available Facilities for Tackling COVID-19

The Institute of Epidemiology, Disease Control and Research (IEDCR) under the Ministry of Health and Family Welfare (MHFW) is responsible for epidemiological and communicable disease research as well as the functioning of disease control programs in Bangladesh. However, at the very beginning of the COVID-19 outbreak, there was very limited support available for testing services. Later, the government of Bangladesh instructed all the government-owned hospitals outside Dhaka to have a “flu- corner”, so that they can collect samples from suspected COVID-19 patients and send those samples to laboratories. Based on the availability of the intensive care unit (ICU), the government declared coronavirus designated hospitals. People with critical health conditions should visit those hospitals.

At present, Table 1 reveals that there are 60 COVID-19 testing centers in order to serve approximately 150 million people in the country. This explains that each testing center is designed to serve approximately 2.5 million people with an imprecise sample of 16,000 per day. The scarcity of testing centers per thousands of people at the division level is significant (Table 2). Moreover, the national helpline (109) has been providing a telemedicine service that includes information on precautions, health guidelines, and directives on suspected COVID-19 infections. This helpline is accessible 24/7. Again, for physicians, personal protective equipment (PPE) is unavoidable equipment before attending to any COVID-19 patient. PPE refers to protective clothing, helmets, gloves, face shields, goggles, surgical masks, respirators, and other equipment, and, therefore, it minimizes the spread of infection from the patient to the medical staff. At the very beginning, particularly in March 2020, most of the hospitals were facing shortages of available PPE. However, the situation has improved. Table 1 shows that the MHFW has provided 1,169,728 items of PPE, 3,128,842 masks, and 190,755 sanitizers up to 6 July 2020.

**Figure 4:**
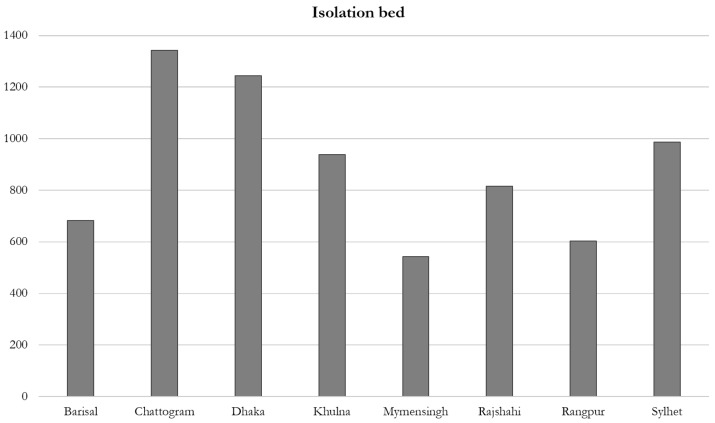
The number of isolation beds in Bangladesh. Data collected from the government websites and field investigation through phone interviews in June 2020.

Again, Figure 4 shows the availability of isolation beds (7157) across the divisions. It can be observed that the Chittagong division has a comparatively significant number of isolation beds (1342), whereas the Mymensingh division has the lowest number of the same (543). However, the number of available beds compared to the number of affected COVID-19 patients (162,417 up to 6 July 2020) is very negligible, i.e., 1 isolation bed is available for every 23 affected patients on average for the country, whereas the situation across the individual division or district level is more competitive. For example, up to 6 July 2020, 46,745 confirmed COVID-19 cases were found in the Dhaka division, but 1 isolation bed was available for every 37 patients.

**Figure 5:**
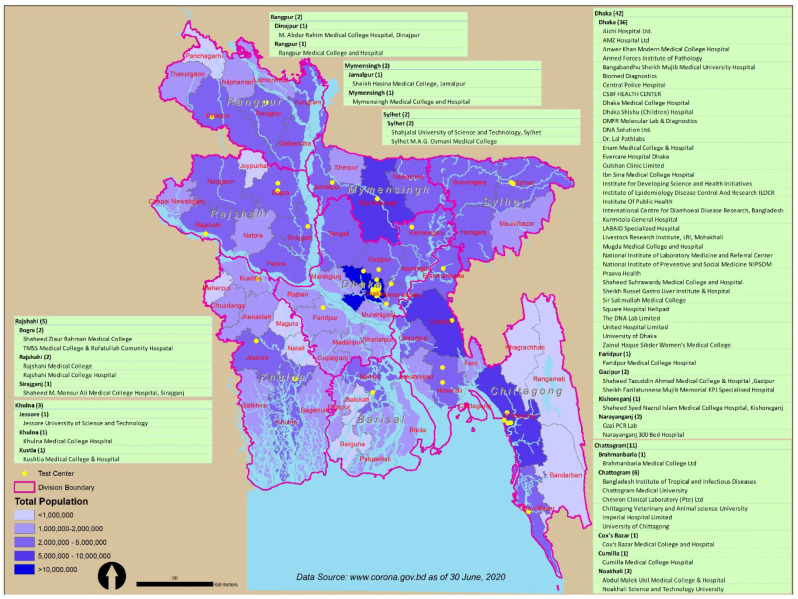
Map of Bangladesh demonstrating the spatial distribution of COVID-19 testing centers (currently operating as of 6 July 2020) in the country. Source: Authors illustration based on the geographic information systems (GIS) and Coronavirus COVID-19 Dashboard, 2020.

According to the recent government data obtained from reliable websites, Figure 5 shows that the major agglomeration of COVID-19 testing centers is located in and around the Dhaka district (39 centers out of 60) in order to serve the maximum population. However, major district centers across the country are embracing a sharp rise in COVID-19 infected people (e.g., Sylhet, Jessore, Khulna, Chittagong, Mymensingh, and Rangpur). Notably, the 60 testing centers across the country may not be in a position to serve the influx of COVID-19-infected people in the coming days and weeks. ‘To get a more accurate picture of the prevalence of COVID-19 in the country at least 10000 people would need to be tested daily’—Nazrul Islam, a Bangladeshi virologist [49].

Moreover, the data in Figure 5 illustrate that in the Barisal division, to date, there is only one testing facility with a testing capacity of approximately 200 per day in order to serve approximately 8 million people. The daily testing rate has remained below 400 per day up to 6 July 2020 in the Barisal, Rangpur, and Sylhet divisions, while the that of the testing facilities in the Chattogram and Dhaka divisions is more than 2000 and 10,000 tests respectively. Additionally, the map reveals population density in the country. The map shows the existing number of testing centers in terms of the total population and density in divisions of Bangladesh. The deep blue-colored area is mostly populated with a high density and has more testing centers than the less blue-colored areas. There is a radical gap in testing centers for COVID-19 among the Dhaka, Barisal, Khulna, and Mymensingh divisions. 

It can be observed that the testing center allocation over the country is not proportionately distributed to the population density; rather, it is allocated in terms of the available hospital services and technical persons and facilities across the country. Most of the testing centers are located in the district-level hospitals and laboratories. Therefore, it was difficult for rural and remotely located people to receive the available testing services, which was also accelerated by the enforced immobility and lockdown activities in the country.

Recent data (as of 12 June 2020) indicate that 17.22% of the tested people who are attending the testing centers sparsely distributed in the country have COVID-19-positive symptoms. Furthermore, according to 2011 Bangladesh Bureau of Statistics (BBS) data (see Table 2), it can be observed that the Rajshahi, Mymensingh, and Dhaka divisions have population densities of more than 1000 people per square kilometer area. However, the COVID-19 testing capabilities are not sufficient to serve the huge population in these named divisions. Table 2 shows that there is a gross difference in test numbers between Dhaka and Barisal, while the conducted test (in millions) is 14,588 and 1309, respectively. Again, despite the notable high density and large population, the number of testing facilities in Mymensingh and Rajshahi is relatively low compared to Dhaka and Chattogram. Barisal has the least testing facilities over the whole country. 

Figure 5. shows that Dhaka and Chittagong city have the majority test centers (53 out of 68), and every day 10,000 and 2000 tests have been conducted, respectively. While the number of people infected with COVID-19 and the number of deaths are rising, the number of testing centers and isolations beds are not rising proportionately. Although the government has started collecting samples in every district town, it is not possible to obtain accurate reports at the right time, as the test facilities are still in the divisional cities, and it is not possible to determine whether the overall death rate is increasing due to lack of testing centers. Although some large districts across the country, namely, Sylhet, Jessore, Khulna, Chittagong Mymensingh and Rangpur, have undergone a rapid rise in COVID-19-positive people, the testing facilities, including isolation beds, medical staff and testing centers, are not sufficient to meet the increasing COVID-19 cases.

### 3.3. Scarcity, Disparity, and Centrality of Testing Facilities 

**Figure 6:**
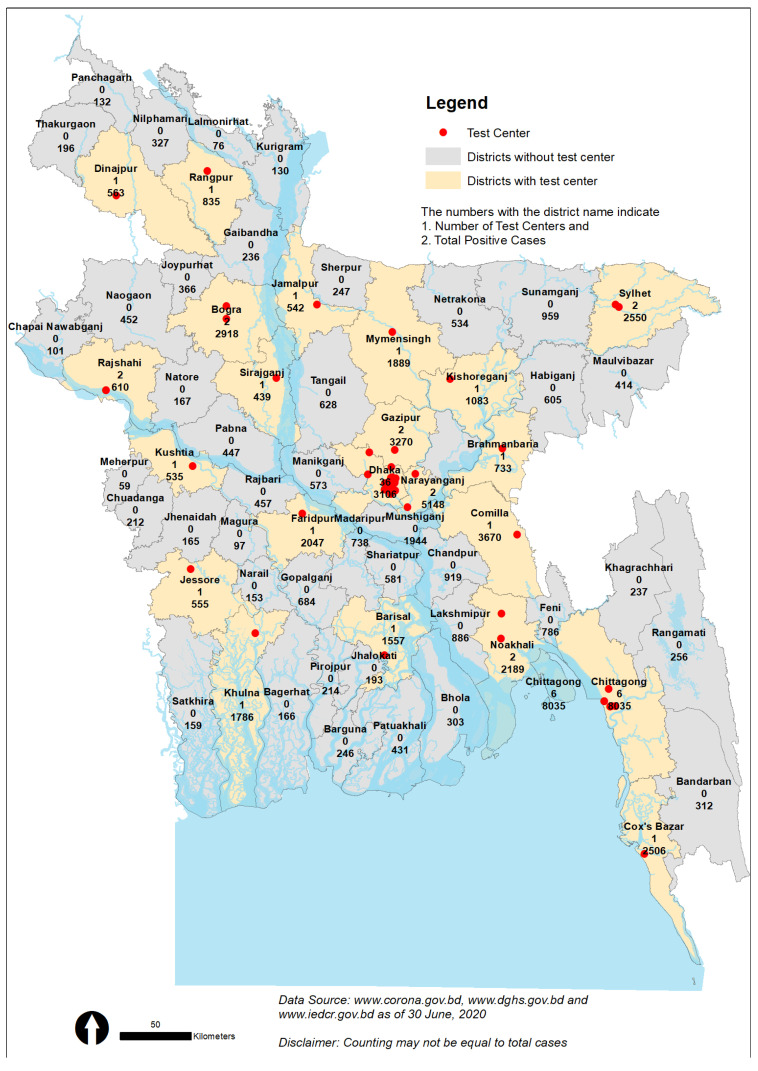
Spatial distribution of testing centers and confirmed COVID-19 cases. Source: Authors illustration based on the GIS and Coronavirus COVID-19 Dashboard, 2020.

All districts in Bangladesh reported at least one COVID-19 cases (Figure 6), but only 30 out of the 64 districts have testing facilities; thus, the regional disparity and scarcity of testing facilities are notably visible. Most of the remote districts have no testing facilities, for example, there are no testing facilities in Bandarban, Rangamati or Khagrachhari, but these districts had 312, 256 and 237 confirmed COVID-19 cases up to 30 June 2020, respectively. Notably, in the Khulna and Barisal divisions, there are only three testing facilities (Khulna-1, Barisal-1, and Kustia-1), where the total number of reported cases is 6831. Again, the Netrokona and Sunamgonj districts have no testing facilities but had 534, and 959 COVID-19-positive cases, respectively. Correspondingly, there is no testing facility in the Nilphamari, Panchagarh, Thakurgaon, Naogaon, or Natore districts, although the numbers of COVID-19-positive cases in these cities are also alarming and may cause massive outbreaks in the northwestern region. A health practitioner working in a remote hospital in Khulna stated, ‘The health system is not well equipped to serve all the population living in the country’.

A similar situation is observed in other districts outside the capital city Dhaka, where the majority of the testing centers (42 out of 68) are located. It is also evident from Figure 6 that almost every district has a significant number of confirmed cases, and that excessive spread is possible due to lack of proper testing. In fact, the remote areas in the country have become a danger zone for silent COVID-19 infections as they are deprived of sufficient testing facilities. It is, therefore, very urgent to intensify the testing facilities in order to identify positive cases, and social distancing and home isolation must also be enforced. If this is not achieved, the whole country may become a red zone on the world map in terms of coronavirus patients, which subsequently will bring massive suffering to the whole country.

After consulting with several COVID-19 test centers over the telephone, we received the above information presented in Table 3. Note that we conducted telephone interviews with maximum of five centers in each division in order to report the average days required to receive the results of COVID-19 tests in hand from dropping the sample off at the center. It would be worthwhile to mention that almost all the reported COVID-19 testing centers were operating under the umbrella of the Ministry of Health, the prime government organization directing public health in the country. However, some private clinics were also involved in testing COVID-19 results, but these are not reported in the mainstream information portal.

**Figure 7:**
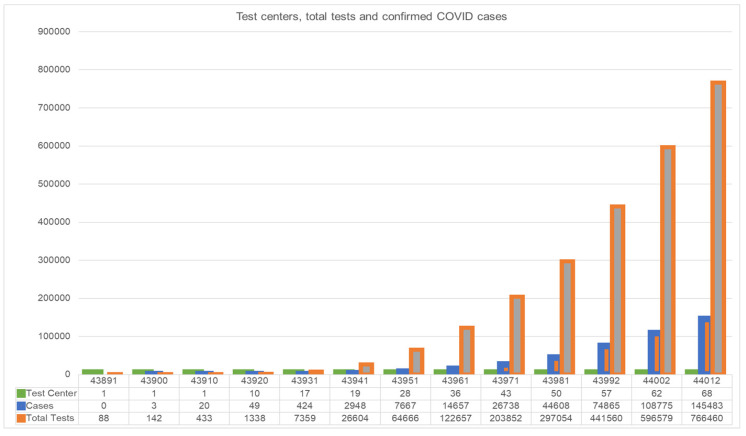
Temporal variation of testing centers, test cases and confirmed COVID-19 cases.

However, while the government of Bangladesh has gradually been improving testing facilities, the progression of the testing facilities compared to the reported cases is very slow and inadequate (Figure 7). For example, only one testing center was in operation on 1 March 2020, while there are 68 testing centers in operation as of 28 June 2020, with 766,460 tests conducted in total. 

Indeed, the entire testing facility for COVID-19 is very centralized and based on district-level hospitals, which may take more than 24 h to collect the samples from rural patients and more than five days to obtain test reports. However, those who are socially reputed, wealthy and have good relations with doctors or nurses involved in the healthcare facilities are being prioritized in terms of receiving tests and reports. Thus, nepotism- and favoritism-type behavior of medical practitioners often discriminates against the poor and underprivileged. Therefore, many poor and disadvantaged patients remain undetected despite the possibility that they are COVID-19 positive. This inequality in the health system of the country results in a questionable process of testing COVID-19 patients, which includes lack of robust screening and testing, and also treatment facilities. Thus, this exacerbates the spread of COVID-19 infection which can grow exponentially, reaching a double in numbers within a week due to symptoms often not being visible. However, a local politician said, ‘Amid the COVID-19 crisis, the measures taken by the government under the leadership of the prime minister are commendable, and steps were taken in the right direction, for example shutting down the business and educational institutions, and introduce for a pro-active lock-down’( based on an interview was conducted on 31 May 2020 with a local ward commissioner in Dhaka city corporation). However, all these arrangements did not receive major attention from the people, as most the country’s population are laborers, and due to the large-scale lock-down, it has become challenging for them to make a living.

## 4. Discussion

### 4.1. Why Do We Need to Test Widely in Diverse Geographic Locations?

Testing is crucial to identify geographically where the infection is and where it is not, and the knowledge gained helps to direct the public health effort in order to combat community spread. Consequently, testing helps to allocate resources where needed, which is considered one of the fundamental disease-surveillance factors. We may not begin to control (e.g., declaring state of emergency, lockdown, or other needed measures) the pandemic unless we know where it is happening and how many cases are confirmed against a given population density. While it may seem obvious that a densely populated area like Dhaka city is likely to have a higher infection rate than the remote countryside, it may require sufficient evidence that will appear with the number of performed tests [50]. Furthermore, testing saves time and equipment allocations in the hospitals upon the identification of who is or is not infected with the virus. Besides, if a particular geographic location is showing a trend of less infected cases, doctors, nurses, personal protective types of equipment (PPE), and ventilators can be mobilized from that region to another where the number of cases is at its peak. Physical distancing, a now-ubiquitous term, may be ensured with strict measures where there are more cases found geographically [51]. Testing provides updated data instantaneously in order to make precise predictions of resource allocation and decisions regarding restricting people from going outside in order to combat the community spread of the virus.

### 4.2. Recommendations for Prioritizing Spatial Decentralization of Testing Capabilities

The South Korean experience regarding mitigation of COVID-19 suggests that large-scale testing is decisive and isolates the geographically infected area that can halt the community spread. However, the case of South Korea may be challenging to employ in Bangladesh because of two primary reasons: the sheer scale at which Bangladesh needs to test surpasses its test kits and lab resources. Secondly, the pandemic experience is new to the policymakers of the country. Thus, government decisions may change within short notice, and availability of testing kits and instruments that can be deployed immediately to all testing centers countrywide may be scarce. As a result, some necessary strategies may help to promote the testing ability of the existing centers at different geographic locations while introducing new testing facilities in order to serve more people spatially throughout the country. For this, the government may plan to introduce some additional short-term and long-term policies, assuming several critical strategies are already in place as:

(i) Enhancing testing capacity at all geographic locations based on population density by removing regulatory and trading barriers;
(ii) Stipulating hospital resources in the worst affected geographic locations so that people may receive necessary treatments immediately when required;
(iii) Encouraging the decentralization of testing facilities into the more local area so that people do not need to travel to other cities, as this may spread the virus quickly in and out of the facility zones;
(iv) Using cell phones to survey, inform, and pre-screen symptoms related to COVID-19 and advising citizens to attend nearby testing facilities where there are ample cases;
(v) If there are shortages of a hospital, clinics, and testing facilities, the government may make a requisition of schools and similar buildings while repurposing them as testing and quarantine facilities throughout the country; the usages of primary schools as cyclone/emergency shelters during cyclone hazard in the coastal area of Bangladesh is likely [33].
(vi) Rapidly scale up the production and distribution of masks at different geographic locations and inspire people to wear them in public places;
(vii) Collaborate with and enhance networks among the testing facilities so that they can share their resources according to the needs of different geographic locations;
(viii) Encourage volunteers to support local-level facilities in order to enhance testing capacity. However, necessary protective measures are highly recommended to the volunteers so that they may not work as an agent of community spread into their local neighborhoods;
(ix) Update the database every day at all testing facilities. This will demonstrate the need for additional resources at different locations. Moreover, the local government may take additional regulatory measures (lockdown in any specific area for a few days) to contest community spread locally;
(x) A critical recommendation is to introduce COVID-19 testing centers in each district geographically (i.e., at district headquarter) in order to increase testing capacities at the local level. This will ensure understanding of the worst-affected areas, and future strategies can be taken immediately upon data availability of infected patients.
(xi) Providing relief aid for poor people where there are more COVID-19-infected people in order to maintain a home isolation period of 2 weeks.

Moreover, policymakers and public health workers should work side by side and build a strong collaboration network to report up-to-date information for local citizens so that people are fully aware of their area and can take precautionary measures immediately. Additionally, we recommend that more testing facilities be introduced considering the population density and remoteness of areas where people do not have easy access to test themselves for COVID-19 virus infection. Furthermore, all major stakeholders should come forward together as a nation under this pandemic to help each other in order to protect their communities.

## 5. Conclusions

Given China’s proximity and a large migrant population living in extensive-outbreak countries like Italy, the Bangladeshi Ministry of Health and Family Welfare should have taken preventive measures when the Chinese government first spread the word about the deadly virus. Unfortunately, valuable time was wasted. However, after a slow start, the government has begun to show signs of urgency by improving the overall facilities for dealing with COVID-19. Notably, the government has imposed social distancing and emergency lockdown, and the local administrations have been told to punish violators of the home quarantine rules in line with Section 269 of the Penal Code. The testing facilities have been slowly established in other locations across the country. It should be kept in mind that the country has a considerable number of governance issues, such as lack of coordination, accountability, healthy decision-making and command protocols. Besides, the poor monetary support, irregularities in distribution, and inadequate preparedness in the health sector, as well as poor health infrastructure together may shape the overall COVID-19 situation in Bangladesh.

Ultimately, the global COVID-19 pandemic has tested the health systems in the world’s wealthiest to developing countries. However, the damages and losses in developing and emerging countries, like Bangladesh, due to the timely implementation of preventing measures are unimaginable. Particularly, the majority of Bangladeshi citizens depend on daily wages and are served by a weak public health system, and, therefore, they are economically too weak to spend money on purchasing COVID-19-related safety equipment, such as masks, sanitizers, etc. Thus, they may be extremely vulnerable under the condition of the uncontrolled spread of COVID-19. Therefore, testing facilities need to be made available across both public and private medical centers, and the government should commit to identifying the community spread of COVID-19 by increasing the number of testing facilities. Thus, the larger the total population, the larger the number of testing centers that should be set up at the district level. This paper has made clearer that Bangladesh’s response to the threat of COVID-19, particularly for testing support, is inadequate. Therefore, the government should strengthen its preparation for testing, screening, isolation, and medical facilities in order to provide better services for tackling COVID-19 as well as future pandemic situations.

However, challenges exist in the form of policies and programs; motivating diverse citizens in understanding the context; ensuring social/physical distances, self-isolation, and self or family quarantine; and supplying relief aid to several communities in dire need. Moreover, with a small amount of resources available to be dispatched, local government organizations started to employ policy strategies in order to prevent community spread and decrease the number of affected people through awareness programs. In order to ensure quality services in healthcare, Bangladesh requires advanced technical assistance in hospitals, and the government should follow the health management practices of developed countries in the health sector.

## Figures and Tables

**Table 1:** Service facilities for mitigating COVID-19.

Particulars	Details	Values
Centers and helplines	No. of testing centers	68
	National call centers	330
	Health portal	16263
	National helpline	109
Category of medical staff	Ayurvedic Medicine Centers (AMC)	2
	Dental Surgeon	6
	Field Staff	168
	Medical Technologist	208
	Nurse	824
	Other (not elsewhere classified)	260
	Physician	990
	Support Staff	566
Total number of facilities available up to 6 July 2020	Apron/gown	76,301
	Gloves—examination	490,551
	Gloves—surgical	472,472
	Head/face/eye shield	1,234,726
	Masks	3,128,842
	Personal Protective Equipment (PPE )kit	1,169,728
	Sanitizer	190,755
	Shoe protector	66,809

Source: Coronavirus COVID-19 Dashboard, 2020.

**Table 2:** Population density and testing facilities of COVID-19.

Division	Area	Population 2011 BBS	Density 2011 BBS	No. of Testing Facilities	People per Testing Center	Test on June 12 2020	Total Test up to June 12 2020	Tests in Million
Barisal	13,225.2	8,325,666	613	1	8,325,666	234	10,900	1309
Chattogram	33,908.6	29,145,000	831	11	2,649,545	2917	84,281	2892
Dhaka	20,593.7	36,433,505	1751	42	867,464	11,850	531,488	14588
Khulna	22,284.2	15,687,759	699	3	5,229,253	735	29,781	1898
Mymensingh	10,584.1	11,370,000	1074	2	5,685,000	998	33,426	2940
Rajshahi	18,153.1	18,485,858	1007	5	3,697,172	846	31,694	1714
Rangpur	16,185.0	15,787,758	960	2	7,893,879	376	24,540	1554
Sylhet	12,635.2	9,807,000	779	2	4,903,500	470	20,350	2075
Total	147,569.1	145,042,546	983	68	2,132,979	18,426	766,460	5284

Source: Bangladesh Bureau of Statistics (BBS), and Coronavirus Dashboard 2020.

**Table 3:** The average duration of COVID-19 test results in eight different divisions.

Division	The Average Number of Days to Receive COVID-19 Test Results
Barisal	8 days
Chattogram	5 days
Dhaka	5 days
Khulna	10 days
Mymensingh	6 days
Rajshahi	6 days
Rangpur	7 days
Sylhet	5 days

Source: Government of Bangladesh, Corona Virus Dashboard 2020.

## References

[1] John Hopkins University (2020). Covid-19 Dashboard by the Center of System Sciences and Engineering at John Hopkins University [Internet]. https://coronavirus.jhu.edu/map.html.

[2] Rodríguez-Morales A.J., Bonilla-Aldana D.K., Tiwari R., Sah R., Rabaan A.A., Dhama K. (2020). COVID-19, an Emerging Coronavirus Infection: Current Scenario and Recent Developments—An Overview. J. Pure Appl. Microbiol..

[3] Collins A.E. (2018). Advancing the Disaster and Development Paradigm. Int. J. Disaster Risk Sci..

[4] Kappes M.S., Papathoma-Köhle M., Keiler M. (2012). Assessing physical vulnerability for multi-hazards using an indicator-based methodology. Appl. Geogr..

[5] Yang P., Wang X. (2020). COVID-19: A new challenge for human beings. Cell. Mol. Immunol..

[6] Velavan T.P., Meyer C.G. (2020). The COVID-19 epidemic. Trop. Med. Int. Health.

[7] Hsiang S., Allen D., Annan-Phan S., Bell K., Bolliger I., Chong T., Druckenmiller H., Huang L.Y., Hultgren A., Krasovich E. (2020). The effect of large-scale anti-contagion policies on the COVID-19 pandemic. Nature.

[8] Bedford J., Enria D., Giesecke J., Heymann D.L., Ihekweazu C., Kobinger G., Lane H.C., Memish Z., Oh M.-D., Sall A.A. (2020). COVID-19: Towards controlling of a pandemic. Lancet.

[9] Watkins J. (2020). Preventing a COVID-19 pandemic. BMJ.

[10] Nicola M., O’Neill N., Sohrabi C., Khan M., Agha M., Agha R. (2020). Evidence based management guideline for the COVID-19 pandemic—Review article. Int. J. Surg..

[11] Cucinotta D., Vanelli M. (2020). WHO Declares COVID-19 a Pandemic. Acta Biomed..

[12] Islam M.S., Rahman K.M., Sun Y., Qureshi M.O., Abdi I., Chughtai A.A., Seale H. (2020). Examining the current intelligence on COVID-19 and infection prevention and control strategies in health settings: A global analysis. Infect. Control Hosp. Epidemiol..

[13] Krzysztofik R., Kantor-Pietraga I., Spórna T. (2020). Spatial and functional dimensions of the COVID-19 epidemic in Poland. Eurasian Geogr. Econ..

[14] Huang R., Liu M., Ding Y. (2020). Spatial-temporal distribution of COVID-19 in China and its prediction: A data-driven modeling analysis. J. Infect. Dev. Ctries..

[15] Chen S., Li Q., Gao S., Kang Y., Shi X. (2020). Mitigating COVID-19 outbreak via high testing capacity and strong transmission-intervention in the United States. MedRxiv.

[16] Chen Y., Jiao J., Bai S., Lindquist J. (2020). Modeling the Spatial Factors of COVID-19 in New York City. SSRN Electron. J..

[17] Algaissi A.A., Alharbi N.K., Hassanain M., Hashem A.M. (2020). Preparedness and response to COVID-19 in Saudi Arabia: Building on MERS experience. J. Infect. Public Health.

[18] Ruktanonchai A.N.W., Floyd J.R., Lai S., Ruktanonchai C.W. (2020). Assessing the impact of coordinated COVID-19 exit strategies across Europe Affiliations. Science.

[19] Raoofi A., Takian A., Sari A.A., Olyaeemanesh A., Haghighi H., Aarabi M. (2020). COVID-19 Pandemic and Comparative Health Policy Learning in Iran. Arch. Iran. Med..

[20] Ihekweazu C., Agogo E. (2020). Africa’s response to COVID-19. BMC Med..

[21] Pedrosa N.L., de Albuquerque N.L.S. (2020). Spatial analysis of COVID-19 cases and intensive care beds in the state of Ceará, Brazil. Cienc Saude Coletiva.

[22] Fanelli D., Piazza F. (2020). Analysis and forecast of COVID-19 spreading in China, Italy and France. Chaos Solitons Fractals.

[23] Shim E., Tariq A., Choi W., Lee Y., Chowell G. (2020). Transmission potential and severity of COVID-19 in South Korea. Int. J. Infect. Dis..

[24] Cohen J., Kupferschmidt K. (2020). Countries test tactics in ‘war’ against COVID-19. Science.

[25] Desmet K., Wacziarg R. (2020). Understanding Spatial Variation in Covid-19 across the United States [Internet]. https://www.anderson.ucla.edu/faculty_pages/romain.wacziarg/downloads/2020_covid.pdf.

[26] Karaye I.M., Horney J.A. (2020). The Impact of Social Vulnerability on COVID-19 in the U.S.: An Analysis of Spatially Varying Relationships. Am. J. Prev. Med..

[27] Mohammad S., Karim T., Alam S. (2020). The Health Care System in Bangladesh: An Insight into Health Policy, Law and Governance Good Governance in Healthcare. Aust. J. Asian Law.

[28] Mahmood S.A.I. (2012). Health systems in Bangladesh. Health Syst. Policy Res..

[29] Corburn J., Vlahov D., Mberu B., Riley L., Caiaffa W.T., Rashid S.F., Ko A.I., Patel S., Jukur S., Martínez-Herrera E. (2020). Slum Health: Arresting COVID-19 and Improving Well-Being in Urban Informal Settlements. J. Urban Hered..

[30] Truelove S., Abrahim O., Altare C., Lauer S.A., Grantz K.H., Azman A.S., Spiegel P. (2020). The potential impact of COVID-19 in refugee camps in Bangladesh and beyond: A modeling study. PLoS Med..

[31] Kumar J., Sahoo S., Bharti B., Walker S. (2020). Spatial distribution and impact assessment of COVID-19 on human health using geospatial technologies in India Spatial distribution and impact assessment of COVID-19 on human health using geospatial technologies in India. Int. J. Multidiscip. Res. Dev..

[32] Chakraborty I., Maity P. (2020). COVID-19 outbreak: Migration, effects on society, global environment and prevention. Sci. Total. Environ..

[33] Mallick B. (2014). Cyclone shelters and their locational suitability: An empirical analysis from coastal Bangladesh. Disasters.

[34] Hamidi S., Sabouri S., Ewing R. (2020). Does Density Aggravate the COVID-19 Pandemic?. J. Am. Plan. Assoc..

[35] Binns C., Low W.Y., Kyung L.M. (2020). The COVID-19 Pandemic: Public Health and Epidemiology. Asia-Pac. J. Public Health.

[36] Julka-Anderson N. (2020). How COVID-19 is testing and evolving our communication skills. J. Med. Imaging Radiat. Sci..

[37] World Health Organization (2020). Preparedness, Prevention and Control of Coronavirus Disease (COVID-19) for Refugees and Migrants in Non-Camp Settings.

[38] Hossain M., Hossain S., Uddin M. (2017). Renewable energy: Prospects and trends in Bangladesh. Renew. Sustain. Energy Rev..

[39] Timsina J., Wolf J., Guilpart N., van Bussel L.G., Grassini P., van Wart J., Hossain A., Rashid H., Islam S., van Ittersum M. (2018). Can Bangladesh produce enough cereals to meet future demand?. Agric. Syst..

[40] United Nations (2015). United Nations Department of Economic and Social Affairs [Internet]. http://esa.un.org/wpp/unpp/panel_population.htm.

[41] Call M.A., Gray C., Yunus M., Emch M. (2017). Disruption, not displacement: Environmental variability and temporary migration in Bangladesh. Glob. Environ. Chang..

[42] (2015). National Food Policy Capacity Strengthening Programme. Adapting Social Safety Net Programs to Climate Change Shocks: Issues and Options for Bangladesh.

[43] Anwar B., Xiao Z., Akter S., Rehman R.U. (2017). Sustainable Urbanization and Development Goals Strategy through Public–Private Partnerships in a South-Asian Metropolis. Sustainability.

[44] Domman D., Chowdhury F., Khan A.I., Dorman M.J., Mutreja A., Uddin M.I., Paul A., Begum Y.A., Charles R.C., Calderwood S.B. (2018). Defining endemic cholera at three levels of spatiotemporal resolution within Bangladesh. Nat. Genet..

[45] Nissan H., Burkart K., de Perez E.C., van Aalst M., Mason S.J. (2017). Defining and Predicting Heat Waves in Bangladesh. J. Appl. Meteorol. Clim..

[46] Parnini S.N. (2006). Civil Society and Good Governance in Bangladesh. Asian J. Politi. Sci..

[47] Doza B., Shammi M., Bahlman L., Islam A.R.T., Rahman M. (2020). Psychosocial and Socio-Economic Crisis in Bangladesh Due to COVID-19 Pandemic: A Perception-Based Assessment. Front. Public Health.

[48] Islam S.D.-U., Doza B., Khan R.M., Haque A., Mamun M.A. (2020). Exploring COVID-19 stress and its factors in Bangladesh: A perception-based study. Heliyon.

[49] Monjur M.R., Hassan Z. (2020). Early phases of COVID-19 management in a low-income country: Bangladesh. Infect. Control. Hosp. Epidemiol..

[50] Islam S., Ira J.I., Kabir K.A., Kamrujjaman M. (2020). COVID-19 Epidemic Compartments Model and Bangladesh. Preprints.

[51] Ramachandran S. The COVID-19 Catastrophe in Bangladesh, The Diplomat, April 29. https://thediplomat.com/2020/04/the-covid-19-catastrophe-in-bangladesh/.

